# Textbook Outcome After Major Liver Resection for Primary and Secondary Liver Tumors at Specialized German Hepatobiliary Centers: Analysis of the StuDoQ Liver Registry

**DOI:** 10.1245/s10434-025-17866-w

**Published:** 2025-08-04

**Authors:** Jan Heil, Svenja Sliwinski, Jan D’Haese, Josef Fangmann, Stefan Farkas, Robert Grützmann, Matthias Glanemann, Jörg C. Kalff, Sören T. Mees, Arianeb Mehrabi, Christoph Michalski, Johann Pratschke, Christoph Reissfelder, Maximilian Schmeding, Matthias Schwarzbach, Gregor A. Stavrou, Jens Werner, Carsten Klinger, Heinz Buhr, Wolf O. Bechstein, Andreas A. Schnitzbauer, for the DGAV StuDoQ-Registry

**Affiliations:** 1 Department of General and Visceral Surgery, Ulm University Hospital, Ulm, Germany; 2 Department of General, Visceral, Transplant and Thoracic Surgery, University Hospital Frankfurt, Goethe University Frankfurt/Main, Frankfurt/Main, Germany; 3 Department of General, Visceral, and Transplantation Surgery, Hospital of the LMU Munich, Ludwig- Maximilians- Universität (LMU), Munich, Germany; 4 Chirurgische Arbeitsgemeinschaft für Leber-, Galle- und Pankreaserkrankungen der Deutschen Gesellschaft für Allgemein- und Viszeralchirurgie, Berlin, Germany; 5 KRH Klinikum Siloah, Liver Center Hannover (LCH), Hannover, Germany; 6 Department of Surgery, St. Josefs- Hospital, Wiesbaden, Germany; 7 Department of General and Visceral Surgery, Friedrich- Alexander- University (FAU) Erlangen- Nuernberg, Erlangen, Germany; 8 Department of General Surgery, Visceral-, Vascular- and Pediatric Surgery, Saarland University Medical Center, Homburg, Germany; 9 Department of Surgery, University Hospital Bonn, Campus 1, Bonn, Germany; 10 Department of General and Visceral Surgery, Dresden- Friedrichstadt General Hospital, Dresden, Germany; 11 Department of General, Visceral, and Transplantation Surgery, Heidelberg University Hospital, Heidelberg, Germany; 12 Charité- Universitätsmedizin Berlin, Corporate Member of Freie Universität Berlin and Humboldt- Universität zu Berlin, Campus Charité Mitte and Campus Virchow- Klinikum, Berlin, Germany; 13 Department of Surgery, Medical Faculty Mannheim, Universitätsmedizin Mannheim, Heidelberg University, Mannheim, Germany; 14 Department of Surgery, Klinikum Dortmund, University Hospital of the University Witten/Herdecke, Witten, Germany; 15 Department of General and Visceral Surgery, Klinikum Frankfurt Höchst, Frankfurt, Germany; 16 Department of General, Visceral and Thoracic Surgery, Surgical Oncology Klinikum Saarbruecken, Saarbruecken, Saarland Germany; 17 StuDoQ Gremium Deutsche Gesellschaft für Allgemein- und Viszeralchirurgie, Berlin, Germany; 18 Knappschaft Kliniken Universitätsklinikum Bochum, Ruhr-Universität-Bochum, Klinik für Chirurgie, Bochum, Germany

**Keywords:** Textbook outcome, Outcome quality, Major hepatectomy, Colorectal liver metastasis, Cholangiocarcinoma, Hepatocellular carcinoma

## Abstract

**Background:**

Textbook outcome (TO) represents the most desirable outcome of surgical quality and care. This study examined the TO of cholangiocarcinoma (CCC), hepatocellular carcinoma (HCC), and colorectal liver metastasis (CRLM) from the StuDoQ liver registry as well as factors that may affect the TO.

**Methods:**

All major liver resections (≥ 3 segments) for CCC, HCC, and CRLM entered in the multicentric StuDoQ liver registry between 2019 and 2022 were retrospectively analyzed. TO was defined by the absence of intraoperative incidents ≥ grade 2 (Oslo criteria), postoperative bile leakage and posthepatectomy liver failure (B/C, ISGLS criteria), major complications (Dindo–Clavien ≥ 3A), 90-day readmission, and mortality rate as well as tumor-free resection margin. Pre- and intraoperative factors that may influence TO were assessed by multivariable analyses.

**Results:**

In 30 participating centers, a total of 1082 major liver resections were performed for CCC (*n* = 396, 36%), HCC (*n* = 214, 20%), and CRLM (*n* = 472, 44%). TO was achieved in 470 (43%) cases, most often in CRLM (51%). Major complications and intraoperative incidents ≥ 2 were the most frequent limiting factors to achieve TO. Anemia (odds ratio (OR) 0.63, 95% confidence interval (CI) 0.47–0.85), simultaneous resection procedures to the liver resection (OR 0.56, 95% CI 0.36–0.88), hyperbilirubinemia (OR 0.53, 95% CI 0.34–0.83), and cholangitis (OR 0.51, 95% CI 0.28–0.94) were identified as modifiable risk factors preventing achievement of a TO.

**Conclusion:**

TO after major liver resection was achieved in less than 50% of cases in certified and high-volume HPB centers. Preoperative risk factors were identified that may allow to improve outcome quality.

**Supplementary Information:**

The online version contains supplementary material available at 10.1245/s10434-025-17866-w.

Liver resection is one of the key pillars in the treatment for primary and secondary liver tumors. However, resectability is limited to a small proportion of patients and varies between 13% and 32%,^1–3^ 8% and 37%,^4–6^ and 20% and 30%^7,8^ for cholangiocarcinoma (CCC), hepatocellular carcinoma (HCC), and colorectal liver metastasis (CRLM). Feasibility and surgical success reflected by technical success was a driving force in recent decades. Technical innovations allowed an expansion of criteria for liver resections, which is why more patients with comorbidities and extensive tumor load were considered for surgery.

Additionally, liver resections were expanded to more complex procedures. In some studies, major liver resections were performed in 24–72%.^2,9–11^ Even simultaneous resections to the liver resection and vascular procedures were performed routinely.^12–15^

However, outcome quality has attracted increasing interest in recent years as it reflects patient factors and disease-specific factors, adding to the quality of care, patient satisfaction, and long-term survival. Initially introduced in colorectal surgery, textbook outcome (TO) combines several outcome quality indicators into a single variable and represents the most desirable outcome after surgery.^16^ Various studies have already investigated TO and could also demonstrate improved oncological outcomes by achieving a TO.^17,18^

However, owing to a lack of standardization, a multiplicity of definitions of TO are available, differing significantly in variates considered relevant for TO.^17^ To overcome this limitation, quality indicators defining TO were recently evaluated in a Delphi process by an international expert panel of HPB surgeons.^19^ Seven intra- and postoperative quality indicators were deemed to be crucial for TO: intraoperative incidents, postoperative bile leakage, posthepatectomy liver failure (PHLF), major complications, 90-day readmission for surgical reasons, 90-day mortality rate, as well as complete tumor resection.

In Germany, liver surgery is not restricted to specific centers and there is no regulation on caseload or outcome quality. Analyses of hospital discharge data have shown considerable in-house mortality, which was also shown to be caseload-dependent.^20,21^ The German Society of General and Visceral Surgery (DGAV) established organ-specific registries, the StuDoQ (Studien-, Dokumentations- und Qualitätszentrums) registries, to assess outcome quality and potential risk factors. Centers in Germany can voluntarily participate in registries, and in case of certification are obliged to document in the registry.

On the basis of the StuDoQ liver registry, this study assessed TO for CCC, HCC, and CRLM after major liver resection. Moreover, pre- and intraoperative variables that may influence TO were investigated.

## Methods

### Study Design

This retrospective study was based on the national multicentric StuDoQ liver registry of the DGAV that was launched in January 2019. The registry listed only tertiary centers. Liver resections performed between January 2019 and December 2021 were extracted from the anonymized database. Reporting of data was performed in accordance with the Strengthening the Reporting of Observational Studies in Epidemiology (STROBE) guidelines.^22^

### Participants

A comprehensive dataset of all patients from the StuDoQ liver registry who underwent major liver resection for CCC, HCC, and CRLM was included in this study.

### Variables

The primary endpoint of this study was TO as described by Gorgec et al.^19^ TO was achieved in the absence of intraoperative incidents ≥ grade 2 (Oslo classification), postoperative bile leakage and PHLF (B/C, ISGLS criteria), major complications (≥ 3A Dindo–Clavien), 90-day readmission for surgical reasons, 90-day mortality rate, as well as complete tumor resection (R0).^19,23–26^ According to the Oslo classification, blood loss over the normal range, the need for resection of accidently injured organs, or conversion from minimally invasive to open surgery were classified as grade 2.^23^ Events beyond that causing relevant consequences for the patient were categorized as grade 3. Postoperative bile leakage grade B was considered if the drainage was in place for more than 1 week or if an additional diagnostic or interventional procedure was necessary.^24^ Meanwhile, the need for a relaparotomy was considered as grade C postoperative bile leakage. PHLF grade B was defined by a deviation from the regular clinical management without the need for invasive treatment,^25^ whereas PHLF grade C was defined by the need for intensive care. Secondary endpoints were variables that may influence TO. Since this study included various tumor types that differ considerably from one another in features such as patient collective, comorbidities, and postoperative outcome, subgroup analyses were carried out.

As only the type of resection performed per liver segment, i.e., anatomical versus non anatomical, is captured by the registry, each anatomical and nonanatomical resection was scored as 1 and 0.5, respectively. On the basis of this scoring, all liver resections with a score of ≥ 3 were defined as major liver resection.^27^ Liver resections with a score of < 3 segments were excluded from the study. The Brisbane 2000 terminology was used to classify liver resections performed.^28^ Reconstructions of portal vein, inferior cava vein, or hepatic artery were described as “vascular procedure”. Also, simultaneous resections in addition to the liver resection, i.e., resection of stomach, pancreas, colon, rectum, or diaphragm, were summarized in the variable “multivisceral resection”.

### Data Source and Management

Prospectively maintained data from local electronic health and tumor board records, as well as operative, procedure, and pathology reports, were entered by participating centers into the anonymized StuDoQ liver registry.

### Bias

Potential reporting bias was reduced by systematic comparison with source files in electronic health and tumor board records, and operative, procedure, and pathology reports. StuDoQ documentation requires an institutional data double-check with discharge data of each documented case in the registry, which reduces bias and reflects annual quality audits by the DGAV. Regarding the Oslo criteria,^23^ intraoperative incidents were not specifically captured by the registry, which was why an underestimation could not be ruled out, if intraoperative incidents did not result in conversion, intraoperative blood transfusion, or postoperative complication according to the Dindo–Clavien classification. Since intraoperative blood loss was also not captured by the registry, intraoperative blood transfusion was alternatively assessed. Furthermore, 90-day readmission for surgical reasons has to be ruled to fulfil TO. As the reason for readmission is not captured by the registry, TO might be underestimated. The registry did not distinguish between different types of CCC, i.e., distal, perihilar, and intrahepatic, which is why applicability to individual tumor entities for cholangiocarcinomas is limited. A final bias may be the definition of nonanatomical liver resections as 0.5 per resection and summing those up in case of multiple resections. The classification of major surgeries is predominantly based on anatomical liver resections, and it still has not finally defined whether three^27^ or four segments^29^ or more should be regarded as major liver resections. As a lot of parenchyma-sparing resections are not adequately depicted in the major surgery system, we decided to value nonanatomical resections as 0.5 segments, recognizing that this is only an approximation.

### Statistics

Descriptive data are provided as median with interquartile range (IQR) for nonparametric data and mean with standard deviation (SD) for parametric data. The Kolmogorow–Smirnow test was used to test the distribution of data. To compare groups, the *t*-test and Mann–Whitney *U* test were used for parametric and nonparametric data, respectively. Fisher’s exact test was used for categorical variables. Potential factors that may influence TO were assessed by multivariable logistic regression using the all-in technique. Analyses and graphics were performed using JMP^®^ 15.0 (SAS Institute) and Graph Pad Prism (Graph Pad Software, La Jolla, CA, USA). Two-sided *p*-values <0.05 were considered statistically significant.

## Results

### Participants

**Fig. 1:**
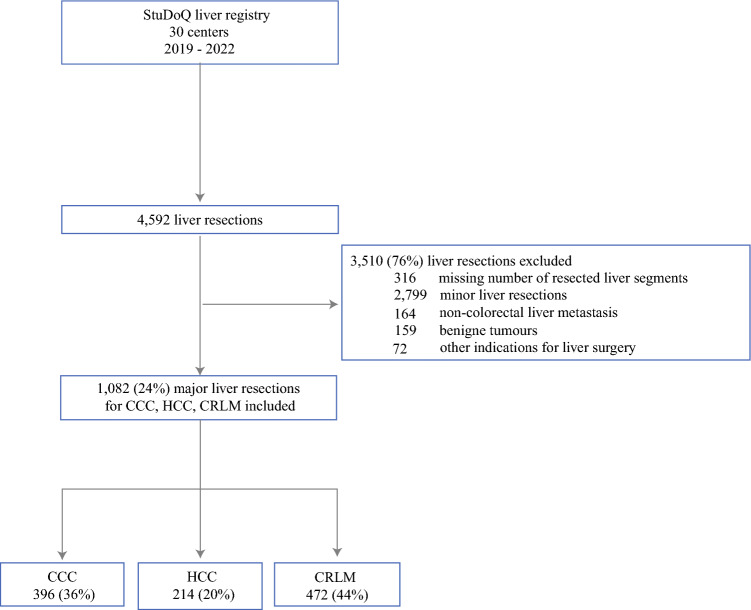
Flowchart

A total of 30 canters participated in the StuDoQ liver registry (Fig. 1). Overall, 4592 liver resections were entered into the database. In 1082 (24%) of those, major liver resections were performed for CCC (*n* = 396, 36%), HCC (*n* = 214, 20%), and CRLM (*n* = 472, 44%).

### Patient Characteristics

The mean age of the entire group was 64 years, and fewer females (39%) than males (61%) underwent major liver resections (Table 1). ASA score of 3 and advanced tumor stage (UICC III/IV) were seen in 58% and 70%, respectively.

**Table 1:** Demographics

Parameter	All	CCC	HCC	CRLM
Patients, *n* (%)	1082	396 (36%)	214 (20%)	472 (44%)
Age [years], mean (SD)	64 (±12)	66 (±11)	67 (±12)	61 (±12)
Sex (female/male), *n* (%)	424/658 (39%/61%)	206/190 (52%/48%)	48/166 (23%/77%)	170/302 (36%/64%)
Height [cm], mean (SD)	172.6 (±8.9)	171.0 (±9.1)	173.2 (±7.8)	173.0 (±9.1)
Weight [kg], mean (SD)	77.3 (±16.5)	76.1 (±15.6)	80.7 (±16.5)	76.8 (±17.0)
BMI [kg/m^2^], mean (SD)	25.9 (±4.8)	26.0 (±4.7)	26.8 (±4.8)	25.4 (±4.8)
*ASA classification*				
ASA 1, *n* (%)	44 (4%)	13 (3%)	7 (3%)	24 (5%)
ASA 2, *n* (%)	390 (36%)	163 (41%)	62 (29%)	165 (35%)
ASA 3, *n* (%)	630 (58%)	212 (54%)	140 (65%)	278 (59%)
ASA 4, *n* (%)	0	0	0	0
ASA 5, *n* (%)	0	0	0	0
Diabetes, *n* (%)	181 (17%)	62 (16%)	70 (33%)	49 (10%)
CHD, *n* (%)	99 (9%)	30 (8%)	32 (15%)	37 (8%)
Cirrhosis, *n* (%)	77 (8%)	15 (4%)	56 (29%)	6 (1%)
COPD, *n* (%)	60 (6%)	23 (6%)	21 (10%)	16 (3%)
Cholangitis, *n* (%)	81 (8%)	56 (14%)	7 (3%)	18 (4%)
Anemia, *n* (%)	385 (36%)	148 (37%)	72 (34%)	165 (35%)
Hyperbilirubinemia, *n* (%)	160 (15%)	114 (29%)	28 (13%)	18 (4%)
*UICC*				
UICC I, *n* (%)	134 (13%)	78 (20%)	56 (27%)	
UICC II, *n* (%)	187 (17%)	111 (29%)	76 (36%)	
UICC III, *n* (%)	241 (23%)	174 (45%)	57 (32%)	
UICC IV, *n* (%)	506 (47%)	24 (6%)	10 (5%)	472 (100%)
*Laboratory results*				
Albumin [g/dl], mean (SD)	4.1 (±0.7)	4.0 (±0.7)	3.9 (±0.7)	4.2 (±0.7)
Bilirubin [mg/dl], median (IQR)	0.5 (0.4–0.8)	0.69 (0.4–1.4)	0.58 (0.4–0.8)	0.40 (0.3–0.6)
Creatinine [mg/dl], median (IQR)	0.8 (0.7–1.0)	0.8 (0.7–1.0)	0.9 (0.7–1.1)	0.8 (0.7–1.0)
Hemoglobin [g/dl], mean (SD)	12.9 (±2.0)	12.8 (±2.0)	13.3 (±2.2)	12.9 (±1.8)
INR, median (IQR)	1.0 (0.9–1.1)	1.0 (0.9–1.1)	1.02 (1.0–1.1)	1.0 (0.9–1.0)

ASA, American Society of Anesthesiologists; BMI, body mass index; CCC, cholangiocellular carcinoma, COPD, chronic obstructive pulmonary disease; CHD, coronary heart disease; CRLM, colorectal liver metastasis; HCC, hepatocellular carcinoma; INR, international normalized ratio; UICC, Union for International Cancer Control

Patients undergoing surgery for CRLM were mostly younger males and tended to have fewer comorbidities (Table 1). In HCC, males (77%) were affected more frequently than females (23%), and the whole group had the highest numbers of comorbidities, including cirrhosis (29%) and diabetes (33%), which was reflected by an increased ASA score of 3 in 65% of patients. Patients with CCC had fewer comorbidities than patients with HCC, but most often preoperative cholangitis (14%) and elevated bilirubin (29%). Additionally, 54% of patients with CCC had advanced tumor stage (UICC III/IV).

### Perioperative Data

Almost all major liver resections of the entire cohort were elective (99%) and performed as one-stage procedures (98%) (Table 2). Portal vein embolization (8%) was most often used as a regenerative maneuver. The majority of resections were open procedures (78%), while laparoscopic surgery (15%) was the predominantly used minimally invasive technique.

**Table 2:** Perioperative data

Parameters	All	CCC	HCC	CRLM
Patients, *n* (%)	1082	396 (36%)	214 (20%)	472 (44%)
Elective surgery, *n* (%)	1067 (99%)	392 (99%)	209 (98%)	466 (99%)
Two-stage hepatectomy, *n* (%)	23 (2%)	5 (1%)	1 (< 1%)	17 (4%)
Portal vein embolization, *n* (%)	87 (8%)	36 (9%)	16 (7%)	35 (7%)
*Resection*				
Open, *n* (%)	843 (78%)	344 (87%)	147 (69%)	352 (75%)
Laparoscopic, *n* (%)	159 (15%)	31 (8%)	46 (21%)	82 (17%)
Robotic, *n* (%)	80 (7%)	21 (5%)	21 (10%)	38 (8%)
Left/right hepatectomy, *n* (%)	217/388 (20%/36%)	108/102 (27%/26%)	46/100 (22%/47%)	63/186 (13%/39%)
Extended left/extended right hepatectomy, *n* (%)	98/230 (9%/21%)	51/108 (13%/27%)	7/33 (3%/15%)	40/89 (9%/19%)
Other resections, *n* (%)	149 (14%)	27 (7%)	28 (13%)	94 (20%)
Biliary procedure, *n* (%)	238 (22%)	199 (50%)	15 (7%)	24 (5%)
Multivisceral resection, *n* (%)	109 (10%)	29 (7%)	15 (7%)	65 (14%)
Vascular procedure, *n* (%)	170 (16%)	118 (30%)	20 (9%)	32 (7%)
Pringle maneuver, *n* (%)	524 (48%)	167 (42%)	112 (52%)	245 (52%)
Intraoperative blood transfusion, *n* (%)	154 (15%)	80 (21%)	33 (16%)	41 (9%)
Operation time [min], mean (SD)	286 (±111)	324 (±120)	252 (±118)	269 (±90)
*Postoperative outcome*				
Intraoperative incidents ≥ 2, *n* (%)	190 (18%)	88 (22%)	43 (20%)	59 (13%)
Resection margin (R0/R1+R2), *n* (%)	804/156 (77%/15%)	284/96 (72%/24%)	175/28 (82%/13%)	345/32 (78%/7%)
PHLF (B/C ISGLS), *n* (%)	56 (5%)	30 (8%)	15 (7%)	11 (2%)
Postoperative biliary leakage (B/C ISGLS), *n* (%)	171 (16%)	89 (23%)	26 (12%)	56 (12%)
Postoperative hemorrhage (B/C ISGLS), *n* (%)	48 (4%)	29 (7%)	9 (4%)	10 (3%)
Major complications (Dindo–Clavien ≥ 3A), *n* (%)	390 (36%)	177 (45%)	76 (36%)	137 (29%)
90-day mortality, *n* (%)	58 (5%)	29 (7%)	16 (8%)	13 (3%)
Textbook outcome (TO), *n* (%)	470 (43%)	132 (33%)	96 (45%)	242 (51%)

ASA, American Society of A;nesthesiologists; BMI, body mass index; CCC, cholangiocellular carcinoma, COPD, chronic obstructive pulmonary disease; CRLM, colorectal liver metastasis; HCC, hepatocellular carcinoma; INR, international normalized ratio; ISGLS, International Study Group of Liver Surgery; PHLF, posthepatectomy liver failure

Extended liver resections (40%), biliary (50%), and vascular (30%) procedures were more frequently performed in CCC than in HCC and CRLM (Table 2). Likewise, the operation time was longer (324 min), and the proportion of intraoperative blood transfusions (21%) was higher. Although minimally invasive procedures were most often used (31%) in HCC, the operation time was the shortest (252 min), which may be due to the low number of extended resections (18%). The Pringle maneuver was equally often used in HCC and CRLM, while fewer red blood transfusions were needed in CRLM (9%) than in HCC (16%). In CRLM, simultaneous resections to the liver resection were performed in 14%, and more often than in CCC (7%) and HCC (7%).

### Postoperative Outcomes

In the entire cohort, complete tumor resection was achieved in 77% (Table 2). Intraoperative incidents (≥ 2) were reported in 18% (*n* = 190). Leading intraoperative incidents ≥ grade 2 were intraoperative blood transfusion (*n* = 154) and conversion from minimally invasive to open surgery (*n* = 36). Major complications and 90-day mortality occurred in 36% and 5%, respectively. Biliary complications (44%) following resections for CCC and posthepatectomy liver failure (14%) were the most common reasons for major complications.

In CRLM, intraoperative incidents (≥ 2) (13%), PHLF (2%), and hemorrhages (3%) were the lowest. Postoperative biliary leakage (23%) and hemorrhage (7%) were most often seen in CCC, and also the major complication rate was higher in CCC (45%) than in HCC (35%) and CRLM (29%). The 90-day mortality was twice as high in CCC (8%) and HCC (7%) than in CRLM (3%).

TO could be evaluated in all patients included in this study and was achieved in 43% of all patients, most often in CRLM (51%), followed by HCC (45%) and CCC (33%) (Table 2). The most limiting factors to achieve a TO were major complications (390, 64%) and intraoperative incidents ≥ grade 2 (190, 31%) (Table 4). Patients failing TO had significantly more often primary liver tumors (TO: 49% vs. no TO: 62%, *p* < 0.001) and differed in tumor stage. Furthermore, anemia (TO: 31% vs. no TO: 39%, *p* = 0.003), hyperbilirubinemia (TO: 8% vs. no TO: 20%, *p* < 0.001), and cholangitis (TO: 4% vs. no TO: 10%, *p* < 0.001) were significantly more common in patients that did not attain TO (Table 3).

**Table 3:** Textbook outcome demographics

Parameters	TO	No TO	
Patients, *n* (%)	470 (43%)	612 (57%)	*p*-Value
Age [years], mean (SD)	64 (±12)	64 (±12)	0.657
Sex (female/male), *n* (%)	183/287 (39%/61%)	241/371 (39%/61%)	0.823
Height [cm], mean (SD)	172.9 (±9.0)	172.3 (±8.8)	0.226
Weight [kg], mean (SD)	77.4 (±15.8)	77.2 (±17.0)	0.757
BMI [kg/m^2^], mean (SD)	25.8 (±4.4)	25.9 (±5.0)	0.704
*ASA classification*			
ASA 1, *n* (%)	26 (6%)	18 (3%)	0.134
ASA 2, *n* (%)	174 (37%)	216 (36%)	
ASA 3, *n* (%)	265 (56%)	365 (60%)	
ASA 4, *n* (%)	0	0	
ASA 5, *n* (%)	0	0	
Diabetes, *n* (%)	82 (18%)	99 (16%)	0.625
CHD, *n* (%)	47 (10%)	52 (9%)	0.479
Cirrhosis, *n* (%)	33 (8%)	44 (8%)	0.955
COPD, *n* (%)	31 (7%)	29 (19%)	0.168
Cholangitis, *n* (%)	18 (4%)	63 (10%)	< 0.001
Anemia, *n* (%)	144 (31%)	241 (39%)	0.003
Hyperbilirubinaemia, *n* (%)	38 (8%)	122 (20%)	< 0.001
*Tumor type*			
CCC, *n* (%)	132 (28%)	264 (43%)	< 0.001
HCC, *n* (%)	96 (21%)	118 (19%)	
CRLM, *n* (%)	242 (51%)	230 (38%)	
*UICC*			
UICC I, *n* (%)	73 (16%)	61 (10%)	< 0.001
UICC II, *n* (%)	72 (15%)	115 (19%)	
UICC III, *n* (%)	68 (15%)	173 (29%)	
UICC IV, *n* (%)	249 (54%)	257 (42%)	
Albumin [g/dl], mean (SD)	4.2 (±0.6)	4.0 (±0.8)	< 0.001
Bilirubin [mg/dl], median (IQR)	0.5 (0.3–0.7)	1 (0.39–1.0)	< 0.001
Creatinine [mg/dl], median (IQR)	0.9 (0.7–1.0)	1.0 (0.7–1.0)	0.723
Hemoglobin [g/dl], mean (SD)	13.2 (±1.8)	12.8 (±2.1)	0.002
INR, median (IQR)	1.0 (1.0–1.1)	1 (0.9–1.1)	0.189

ASA, American society of anesthesiologists; BMI, body mass index; CCC, cholangiocellular carcinoma, COPD, chronic obstructive pulmonary disease; CHD, coronary heart disease; CRLM, colorectal liver metastasis; HCC, hepatocellular carcinoma; INR, international normalized ratio; UICC, Union for International Cancer Control

Following open surgery, significantly more patients failed TO (TO: 73% vs. no TO: 81%, *p* = 0.022), while significantly more patients reached TO who underwent minimally invasive surgery (TO: 27% vs. no TO: 19%, *p* = 0.022) (Table 4). Likewise, the Pringle maneuver was significantly more often used in patients who had TO (TO: 53% vs. no TO: 43%, *p* = 0.003). Meanwhile, for liver resections in which multivisceral resections (TO: 8% vs. no TO: 14%, *p* = 0.005), biliary (TO: 13% vs. no TO: 29%, *p* < 0.001) or vascular (TO: 10% vs. no TO: 20%, *p* < 0.001) procedures were performed, significantly more patients did not achieve TO. Also, the operation time was significantly longer in patients not reaching TO (TO: 259 min vs. no TO: 306 min, *p* < 0.001). Additionally, it was investigated whether the TO varied depending on the caseload of participating centers (Supplementary Fig. 1). Categorized into five groups depending on the annual number of liver resections performed, TO was comparable between participating centers.

**Table 4:** Textbook outcome perioperative data

Parameters	TO	No TO	
Patients, *n* (%)	470 (43%)	612 (57%)	*p*-Value
Elective surgery, *n* (%)	460 (99%)	606 (99%)	0.807
Two-stage hepatectomy, *n* (%)	11 (2%)	12 (2%)	0.734
Portal vein embolization, *n* (%)	27 (9%)	60 (16%)	0.021
*Operative procedure*			
Open, *n* (%)	342 (73%)	500 (81%)	0.022
Laparoscopic, *n* (%)	82 (18%)	77 (13%)	
Robotic, *n* (%)	42 (9%)	38 (6%)	
Biliary procedure, *n* (%)	60 (13%)	178 (29%)	< 0.001
Multivisceral resection, *n* (%)	38 (8%)	84 (14%)	0.005
Vascular procedure, *n* (%)	48 (10%)	122 (20%)	< 0.001
Pringle maneuver, *n* (%)	249 (53%)	264 (43%)	0.003
Intraoperative blood transfusion, *n* (%)	0	154 (26%)	< 0.001
Operation time [min], mean (SD)	259 (±95)	306 (±119)	< 0.001
*Postoperative outcome*			
Intraoperative incidents ≥ 2, *n* (%)	0	190 (31%)	< 0.001
Resection margin (R0/R1+R2), *n* (%)	409/0 (88%/0)	394/156 (64%/36%)	< 0.001
PHLF (B/C ISGLS), *n* (%)	0	56 (10%)	< 0.001
Postoperative biliary leakage (B/C ISGLS), *n* (%)	0	171 (28%)	< 0.001
Postoperative hemorrhage (B/C ISGLS), *n* (%)	3 (1%)	45 (7%)	< 0.001
Major complications (Dindo-Clavien ≥3A), *n* (%)	0	390 (64%)	< 0.001
90- day mortality, *n* (%)	0	58 (9%)	< 0.001

ISGLS, International Study Group of Liver Surgery; PHLF, posthepatectomy liver failure

### Factors Influencing Textbook Outcome

**Fig. 2:**
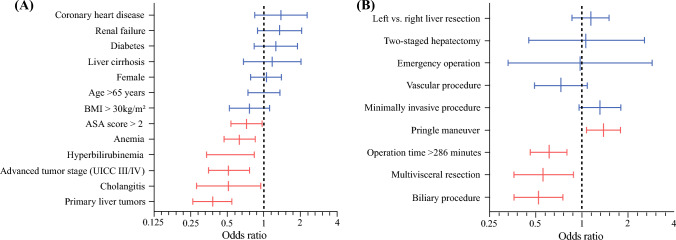
**A** Odds ratios for preoperative factors to achieve TO in the multivariable analysis. **B** Odds ratios for intraoperative factors to achieve TO in the multivariable analysis

On multivariable analysis (MVA) of preoperative variables, primary liver tumors (odds ratio (OR) 0.38, 95% confidence interval (CI) 0.26–0.55), cholangitis (OR 0.51, 95% CI 0.28–0.94), advanced tumor stage (UICC III/IV) (OR 0.51, 95% CI 0.35–0.76), elevated bilirubin (OR 0.53, 95% CI 0.34–0.83), anemia (OR 0.63, 95% CI 0.47–0.85), and ASA score > 2 (OR 0.72, 95% CI 0.54–0.96) were demonstrated to be associated with a significantly lower chance of achieving TO (Fig. 2A; Supplementary Table 1A).

Of the intraoperative variables investigated, biliary procedures (OR 0.52, 95% CI 0.36–0.88), multivisceral resections (OR 0.56, 95% CI 0.36–0.88), and operation time > 286 min (OR 0.61, 95% CI 0.46–0.80) were shown to be significant obstacles to achieving TO (Fig. 2B; Supplementary Table 1B). The Pringle maneuver (OR 1.38, 95% CI 1.07–1.78) was the only variable that was positively associated with achieving TO.

### Subgroup Analyses for CCC, HCC, and CRLM

In CCC, preoperative elevated bilirubin (OR 0.47, 95% CI 0.27–0.84) and anemia (OR 0.50, 95% CI 0.30–0.85) as well as biliary procedures (OR 0.49, 95% CI 0.50–0.83) and prolonged operation time (OR 0.47, 95% CI 0.28–0.77) were demonstrated to be significant obstacles to reaching TO (Supplementary Table 2A, B), while minimally invasive procedures provided a significantly higher chance of achieving TO in CCC (OR 3.26, 95% CI 1.66–6.44).

In HCC, none of the preoperative variables investigated appeared to influence TO (Supplementary Table 3A, B). Of intraoperative variables, the Pringle maneuver was significantly associated with achieving TO (OR 2.53, 95% CI 1.37–4.68), while multivisceral resections were demonstrated to be associated with a lower chance of achieving TO in HCC (OR 0.09, 95% CI 0.01–0.70).

In CRLM, preoperative anemia was associated with a lower chance of achieving TO (OR 0.65, 95% CI 0.42–0.99) as well as prolonged operation time (OR 0.57, 95% CI 0.38–0.84) (Supplementary Table 4A/B).

## Discussion

This is the first study providing TO after major liver resection for CCC, HCC, and CRLM on the basis of the multicentric StuDoQ liver registry that represents 40% of all major liver resections performed in Germany.^20^ TO was achieved in nearly half of all procedures, most frequently in CRLM. Major complications and intraoperative incidents ≥ grade 2 were the most common limiting factors for achieving TO. Primary liver tumors and advanced tumor stage (UICC III/IV) were associated with not obtaining TO. Meanwhile, cholangitis, elevated bilirubin, and anemia were identified as preoperative modifiable risk factors that may allow improving postoperative outcomes. Of intraoperative factors investigated, biliary procedures and prolonged operation time were shown to be associated with a lower chance of reaching TO. Simultaneous multivisceral resections added to liver resection were identified as a major modifiable predictor for not attaining TO and raises the question of whether simultaneous resections should be performed.

A recent study by our group that was based on hospital discharge data from the German Federal Statistic Office revealed an unexpectedly high mortality rate.^20^ The mortality rate was found to be caseload-dependent but also showed differences between the various tumors entities included and the complexity of the procedures performed, as also shown by others.^21^ In particular, in primary liver tumors, simultaneous resections in addition to the liver resection resulted in a notable increase of the mortality rate.^20^

Studies on TO have attracted increased interest in recent years.^17^ One reason may certainly be that TO combines several outcome measurements into a single variable and therefore makes assessment and comparison of outcome quality easier.^11,16^ However, hasty conclusions should be avoided when comparing TOs. Various definitions have been used with significant differences in outcome parameters considered relevant for a TO. While the definition of TO used in this study originates from an international consensus meeting and was elaborated in a Delphi process.^19^ This may allow standardization in upcoming studies. However, the definition also has some weaknesses, as the length of hospital stay is not considered. Complications, such as pneumonia or thromboembolism, can result in prolonged hospital stay or admission to the intensive/intermediate care unit in the worst case, which is a blind spot of the definition and may result in an overestimation of TO. It should also be mentioned that not all parameters of a TO can be achieved under all circumstances, or may have a different meaning under certain circumstances. Positive resection margin (R1/R2) was one of the most common reasons for failing TO in this study. In CRLM, the impact of R1 margins is controversially discussed, and vascular R1 resection margin cannot be seen as technical failure per se.^30,31^ In perihilar cholangiocarcinoma, R1 resection may have limited oncological relevance in the case of nodal metastases and is therefore deliberately accepted.^32,33^ By representing the best theoretically outcome, however, TO may help to identify processes that can be optimized to improve surgical quality and care. ^34^

The TO in this study lies in the mid-range in comparison with other studies that reported TOs between 13% and 84%.^17^ However, as every center needs to fully report their liver resections to the registry when participating, there is a high probability of valid and nonselected data. The variation of TOs may be partially due to the different definitions used, a potential risk of selection, but also to different tumor types included. For CRLM, a TO of 84%^18^ was described, compared with 62% for HCC,^10^ 24% for perihilar,^35^ and 22% for intrahepatic cholangiocarcinoma.^36^ In this study, TO was most often achieved in CRLM (51%), but less often in HCC (45%) than in CCC (33%). Differences in TO were also seen between minor versus major liver resection and open versus minimally invasive surgery. ^17^

Several pre- and intraoperative factors have been identified as impacting TO. Of the preoperative factors investigated, primary liver tumors and advanced tumor stage (UICC III/IV) were identified as significant obstacles, which is in line with other studies that also described non-CRLM, tumor size, number of lesions, bilobar disease, major resections, and major vascular invasion as significant risk factors for not achieving TO,^9,17^ while cholangitis and hyperbilirubinemia were demonstrated to be modifiable preoperative risk factors that therefore may allow to improve outcomes. Cholangitis is known to be associated with increased postoperative morbidity and mortality,^37,38^ which is why biliary stenting or drainage is common practice in patients with obstructive cholangitis. However, in the case of isolated elevated bilirubin without cholangitis, the placement of a stent or drainage is controversially discussed because of concerns about causing infections or cholangitis by stenting or draining the biliary tree.^39,40^ A recent series described biliary drainage as a predictor for not achieving TO, and also increased morbidity due to post-hepatectomy liver failure has been described in patients with external biliary drainage.^14,35^ Unfortunately, this registry does not consider the preoperative placement of a stent or drainage. However, already elevated bilirubin beyond the normal range was considered as hyperbilirubinemia in this study and remained significant in contrast to cholangitis in the subgroup analysis for CCC in the MVA. It can be concluded from this that increased bilirubin is more serious than a potential cholangitis due to a biliary drainage, which is supported by previous studies describing increased morbidity and mortality in patients with increased bilirubin.^38,41^

Anemia was found to be an additional preoperative predictor of not achieving TO, which is in line with previous studies describing an increased rate of morbidity and mortality for preoperative anemia.^42,43^ However, preoperative iron supplementation in iron deficiency-related anemia, which is the most common reason for anemia, was shown to reduce intraoperative red blood cell transfusion and to improve oncological outcomes.^44^

Of the intraoperative factors investigated, biliary procedures and prolonged operation time were also associated with a decreased likelihood of achieving TO. Other studies have also revealed complex liver resections with the need for resection of the posterior segments, major liver resections as well as vascular and biliary reconstructions as significant determinants for failing to achieve TO.^17^ This demonstrates that the present classification of a “major resection” has only limited validity,^27,29^ since the complexity of a liver resection cannot only be described by the extent of resection. Nonanatomical resections of lesions deeper in the parenchyma and close to major vascular structures or reconstructive procedures of the biliary or the vascular system are technically challenging and prone to complications.^45^ In this study, a scoring system was established in which each nonanatomical resection performed per segment was scored 0.5 and considered a “major resection” from a total of 3. This classification is only an approximation that at least considers the performance of nonanatomical and parenchymal-sparing resections, respectively.

Additionally, multivisceral resections were associated with not achieving TO in the MVA for the entire cohort. Although simultaneous resections to the liver resection were predominantly performed in CRLM and significantly more patients undergoing multivisceral resection failed TO, there was no significant association in subgroup analysis for CRLM in the MVA. However, in primarily resectable CRLM, simultaneous resection of the liver metastasis to the colon or rectal tumor is controversially discussed.^15,46^ Simultaneous resections provide the benefit of requiring only a single resection, but may also bear an increased risk, especially when performing a combined rectal and major liver resection. A recent randomized controlled trial investigating simultaneous versus staged resection for CRLM did not find differences in postoperative complications.^47^ However, there was a tendency for more digestive complications following simultaneous resections (28% vs. 13%, *p* = 0.08), and only a 60-day follow-up of surgical complications was provided. Meanwhile, no differences were seen in the oncological outcome after a median follow-up of 46 months. Unfortunately, the StuDoQ liver registry does not provide follow-up longer than 90 days, which is beyond the scope of the registry, and also the cause of complications is not specified. However, a recent study assessed long-term follow-up in CRLM and revealed a significantly improved overall survival of patients who achieved TO (51 vs. 39 months, *p* = 0.013).^18^ Whether patients underwent simultaneous resections is not provided unfortunately, but the study underlines the benefit of achieving TO.

The Pringle maneuver was the only factor that was associated with an increased chance of achieving TO. This observation might be due to the Oslo criteria,^23^ which assess increased intraoperative blood loss, which was evaluated by intraoperative red blood cell transfusions in this study. The Pringle maneuver reduces intraoperative bleeding and the need for blood transfusions by reducing the hepatic inflow, which has been shown to improve postoperative outcomes.^48^

A couple of variables were demonstrated not to have an influence on TO in this study, although it may have been expected. Vascular procedures increase the complexity of liver resections and were shown to negatively affect TO.^17^ Although significant more vascular procedures were performed in patients failing TO, there was no association seen between vascular procedures and TO in the MVA. Cirrhosis, most common in HCC, was shown to not affect TO. Even patients failing TO did not differ regarding the prevalence of cirrhosis in comparison with patients who reached TO, which might be due to a proper preoperative selection of patients undergoing liver resection. Right-sided liver resections in perihilar cholangiocarcinoma were shown to result in worse short- and long-term outcomes compared with left-sided resections.^12^ In this study, no difference was seen in TO between these two approaches, which might be due to the inhomogeneous inclusion of different types of cholangiocarcinoma.

This study has several limitations. First, selection and era bias cannot be excluded owing to the retrospective study design. However, these biases were reduced by a large study size and performing MVAs. Second, multiple definitions of TO are available, which in turn explain the differences of TO achieved as well as the variation of factors impacting TO. Without standardization, comparison between studies is limited. The definition of TO used in this study was elaborated in a Delphi process and considers a comprehensive selection of quality indicators.

In summary, this is the largest series providing TO for primary and secondary liver tumors on the basis of a German multicentric registry. Outcome quality was comparable to international data. Several pre- and intraoperative factors were identified that allow for improved outcome quality. Further studies are warranted to investigate whether improved TO results in an improved oncological outcome.

## Supplementary Information

Below is the link to the electronic supplementary material.

Supplementary file1 (DOCX 27 kb)
