# Supplementary material for: Textbook Outcome After Major Liver Resection for Primary and Secondary Liver Tumors at Specialized German Hepatobiliary Centers: Analysis of the StuDoQ Liver Registry
Source: Ann Surg Oncol. 2025 Aug 4;32(10):7183–94. doi: 10.1245/s10434-025-17866-w (PMC12454482; doi:10.1245/s10434-025-17866-w)
Supplement: Supplementary file 1 — Supplementary file1 (DOCX 27 kb) [file 10434_2025_17866_MOESM1_ESM.docx]

| **Supplementary table 1A** Uni- and multivariable analysis of preoperativ factors for textbook outcome in primary and secondary liver tumors | | | | |
| --- | --- | --- | --- | --- |
| **Variables** | univariable | | multivariable | |
|  | OR (CI) | p-value | OR (CI) | p-value |
| Coronary heart disease | 1.20 (0.79-1.81) | 0.396 | 1.38 (0.84-2.26) | 0.202 |
| Renal failure | 1.25 (0.88-1.77) | 0.218 | 1.34 (0.88-2.05) | 0.168 |
| Diabetes | 1.10 (0.80-1.51) | 0.579 | 1.25 (0.83-1.89) | 0.282 |
| Liver cirrhosis | 0.98 (0.61-1.55) | 0.902 | 1.17 (0.68-2.03) | 0.562 |
| Female | 0.98 (0.77-1.26) | 0.882 | 1.05 (0.78-1.40) | 0.765 |
| Age >65 years | 0.94 (0.74-1.19) | 0.587 | 1.00 (0.74-1.35) | 0.997 |
| BMI >30kg/m^2^ | 0.83 (0.60-1.15) | 0.259 | 0.76 (0.52-1.11) | 0.154 |
| ASA score >2 | 0.85 (0.66-1.09) | 0.194 | 0.72 (0.54-0.96) | **0.024** |
| Anemia | 0.68 (0.53-0.88) | **0.003** | 0.63 (0.47-0.85) | **0.002** |
| Hyperbilirubinemia | 0.35 (0.24-0.52) | **<0.001** | 0.53 (0.34-0.83) | **0.005** |
| Advanced tumor stage (UICC III/IV) | 0.90 (0.69-1.16) | 0.408 | 0.51 (0.35-0.76) | **<0.001** |
| Cholangitis | 0.35 (0.20-0.60) | **<0.001** | 0.51 (0.28-0.94) | **0.032** |
| Primary liver tumors | 0.37 (0.44-0.72) | **<0.001** | 0.38 (0.26-0.55) | **<0.001** |
| ASA: American society of anesthesiologists; BMI: body mass index; CI: confidence interval; OR: odds ratio; UICC: Union for International Cancer Control | | | | |

| **Supplementary table 1B** Uni- and multivariable analysis of intraoperative factors for textbook outcome in primary and secondary liver tumors | | | | |
| --- | --- | --- | --- | --- |
| **Variables** | univariable | | multivariable | |
|  | OR (CI) | p-value | OR (CI) | p-value |
| Left or extended left versus  right or extended right hepatectomy | 1.11 (0.86-1.46) | 0.405 | 1.14 (0.86-1.50) | 0.357 |
| Two-staged hepatectomy | 1.20 (0.52-2.74) | 0.668 | 1.06 (0.45-2.56) | 0.890 |
| Emergency procedure | 1.15 (0.41-3.27) | 0.787 | 0.97 (0.33-2.86) | 0.890 |
| Vascular procedure^*^ | 0.47 (0.33-0.68) | **<0.001** | 0.73 (0.49-1.08) | 0.117 |
| Minimally invasive procedure | 1.55 (1.16-2.07) | **0.003** | 1.31 (0.96-1.79) | 0.090 |
| Pringle maneuver | 1.51 (1.19-1.93) | **<0.001** | 1.38 (1.07-1.78) | **0.015** |
| Operation time  >286 minutes | 0.50 (0.39-0.64) | **<0.001** | 0.61 (0.46-0.80) | **<0.001** |
| Multivisceral resection^+^ | 0.51 (0.33-0.78) | **0.002** | 0.56 (0.36-0.88) | **0.012** |
| Biliary procedure | 0.38 (0.27-0.52) | **<0.001** | 0.52 (0.36-0.75) | **<0.001** |
| CI: confidence interval; OR: odds ratio;  ^*^Vascular procedure: reconstructions of portal vein, inferior cava vein or hepatic artery  ^+^Multivisceral resection: resection of stomach, pancreas, colon, rectum or diaphragm | | | | |
|  | | | | |

| **Supplementary table 2A** Uni- and multivariable analysis of preoperative factors for textbook outcome in CCC | | | | |
| --- | --- | --- | --- | --- |
| **Variables** | univariable | | multivariable | |
|  | OR (CI) | p-value | OR (CI) | p-value |
| Diabetes | 1.43 (0.82-2.50) | 0.205 | 1.89 (0.92-3.90) | 0.084 |
| Coronary heart disease | 1.37 (0.64-2.93) | 0.422 | 1.50 (0.58-3.78) | 0.414 |
| Renal failure | 1.35 (0.75-2.41) | 0.315 | 1.36 (0.68-2.75) | 0.388 |
| Female | 1.40 (0.92-2.13) | 0.118 | 1.34 (0.81-2.21) | 0.249 |
| ASA score >2 | 0.81 (0.53-1.24) | 0.332 | 0.75 (0.46-1.24) | 0.261 |
| Liver cirrhosis | 0.71 (0.22-2.28) | 0.568 | 0.86 (0.48-3.31) | 0.375 |
| Age >65 years | 0.88 (0.58-1.34) | 0.539 | 0.79 (0.52-1.52) | 0.673 |
| Cholangitis | 0.39 (0.19-0.80) | **0.010** | 0.61 (0.27-1.38) | 0.234 |
| BMI >30 kg/m^2^ | 0.74 (0.43-1.28) | 0.277 | 0.52 (0.27-1.01) | 0.052 |
| Anemia | 0.60 (0.38-0.93) | **0.023** | 0.50 (0.30-0.85) | **0.010** |
| Hyperbilirubinaemia | 0.37 (0.22-0.63) | **0.002** | 0.47 (0.27-0.84) | **0.011** |
| ASA: American society of anesthesiologists; BMI: body mass index; CI: confidence interval; OR: odd ratio | | | | |

| **Supplementary table 2B** Uni- and multivariable analysis of intraoperative factors for textbook outcome in CCC | | | | |
| --- | --- | --- | --- | --- |
| **Variables** | univariable | | multivariable | |
|  | OR (CI) | p-value | OR (CI) | p-value |
| Emergency procedure | 2.02 (0.28-14.50) | 0.486 | 1.96 (0.26-14.59) | 0.512 |
| Two-staged resection | 3.05 (0.50-18.46) | 0.226 | 1.82 (0.27-12.23) | 0.540 |
| Pringle maneuver | 1.69 (1.11-2.58) | 0.015 | 1.51 (0.95-2.41) | 0.082 |
| Left or extended left versus  right or extended right hepatectomy | 1.21 (0.79-1.85) | 0.385 | 1.10 (0.69-1.77) | 0.683 |
| Vascular procedure^*^ | 0.49 (0.30-0.80) | **0.005** | 0.87 (0.49-1.54) | 0.637 |
| Multivisceral procedure^+^ | 0.50 (0.20-1.26) | 0.140 | 0.56 (0.21-1.50) | 0.250 |
| Minimally invasive procedure | 3.90 (2.13-7.15) | **<0.001** | 3.26 (1.66-6.44) | **<0.001** |
| Biliary procedure | 0.30 (0.19-0.47) | **<0.001** | 0.49 (0.50-0.83) | **0.008** |
| Operation time >286 minutes | 0.39 (0.25-0.60) | **<0.001** | 0.47 (0.28-0.77) | **0.003** |
| CI: confidence interval; OR: odds ratio  ^*^Vascular procedure: reconstructions of portal vein, inferior cava vein or hepatic artery  ^+^Multivisceral resection: resection of stomach, pancreas, colon, rectum or diaphragm | | | | |

| **Supplementary table 3A** Uni- and multivariable analysis of preoperative factors for textbook outcome in HCC | | | | |
| --- | --- | --- | --- | --- |
| **Variables** | univariable | | multivariable | |
|  | OR (CI) | p-value | OR (CI) | p-value |
| Age >65 years | 1.11 (0.63-1.94) | 0.725 | 1.37 (0.68-2.76) | 0.373 |
| Coronary heart disease | 0.82 (0.38-1.75) | 0.602 | 1.29 (0.53-3.18) | 0.568 |
| Female | 1.31 (0.69-2.50) | 0.417 | 1.16 (0.54-2.51) | 0.704 |
| BMI >30 kg/m^2^ | 1.00 (0.50-2.00) | 0.992 | 1.07 (0.47-2.40) | 0.879 |
| Renal failure | 0.80 (0.40-1.62) | 0.534 | 1.02 (0.45-2.30) | 0.958 |
| Liver cirrhosis | 0.96 (0.51-1.79) | 0.896 | 0.86 (0.42-1.77) | 0.684 |
| Anemia | 0.71 (0.40-1.27) | 0.253 | 0.76 (0.38-1.49) | 0.419 |
| ASA score >2 | 0.75 (0.42-1.34) | 0.340 | 0.67 (0.35-1.31) | 0.242 |
| Hyperbilirubinaemia | 0.55 (0.24-1.29) | 0.169 | 0.67 (0.24-1.84) | 0.438 |
| Diabetes | 0.57 (0.32-1.03) | 0.062 | 0.59 (0.28-1.26) | 0.173 |
| Cholangitis | 0.20 (0.02-1.66) | 0.135 | 0.21 (0.02-1.90) | 0.166 |
| ASA: American society of anesthesiologists; BMI: body mass index; CI: confidence interval; OR: odds ratio; | | | | |

| **Supplementary table 3B** Uni- and multivariable analysis of intraoperative factors for textbook outcome in HCC | | | | |
| --- | --- | --- | --- | --- |
| **Variables** | univariable | | multivariable | |
|  | OR (CI) | p-value | OR (CI) | p-value |
| Left or extended left versus  right or extended right hepatectomy | 1.88 (1.00-3.52) | 0.049 | 1.86 (0.94-3.65) | 0.073 |
| Biliary procedure | 1.08 (0.38-3.10) | 0.884 | 1.63 (0.43-6.17) | 0.473 |
| Operation time >286 minutes | 1.00 (0.54-1.87) | 0.986 | 1.18 (0.58-2.43) | 0.647 |
| Minimally invasive procedure | 1.29 (0.73-2.31) | 0.383 | 0.78 (0.40-1.52) | 0.461 |
| Emergency procedure | 0.82 (0.13-4.98) | 0.825 | 0.79 (0.11-5.54) | 0.809 |
| Vascular procedure^*^ | 0.38 (0.13-1.08) | 0.069 | 0.39 (0.13-1.20) | 0.101 |
| Pringle maneuver | 2.69 (1.54-4.70) | **<0.001** | 2.53 (1.37-4.68) | **0.003** |
| Multivisceral resection^+^ | 0.07 (0.01-0.56) | **0.012** | 0.09 (0.01-0.70) | **0.022** |
| CI: confidence interval; OR: odds ratio;  ^*^Vascular procedure: reconstructions of portal vein, inferior cava vein or hepatic artery  ^+^Multivisceral resection: resection of stomach, pancreas, colon, rectum or diaphragm | | | | |
|  | | | | |

| **Supplementary table 4A** Uni- and multivariable analysis of preoperative factors for textbook outcome in CRLM | | | | |
| --- | --- | --- | --- | --- |
| **Variables** | univariable | | multivariable | |
|  | OR (CI) | p-value | OR (CI) | p-value |
| Liver cirrhosis | 4.67 (0.54-40.36) | 0.161 | 4.37 (0.47-40.82) | 0.195 |
| Renal failure | 1.90 (1.02-3.53) | **0.042** | 1.99 (0.96-4.15) | 0.066 |
| Diabetes | 1.91 (1.03-3.54) | **0.041** | 1.72 (0.82-3,59) | 0.151 |
| Coronary heart disease | 1.43 (0.72-2.84) | 0.301 | 1.35 (0.60-3.04) | 0.465 |
| Age >65 years | 1.20 (0.82-1.75) | 0.350 | 1.09 (0.70-1.68) | 0.709 |
| BMI >30kg/m^2^ | 0.94 (0.56-1.58) | 0.821 | 0.96 (0.52-1.75) | 0.892 |
| Female | 0.86 (0.59-1.25) | 0.425 | 0.92 (0.60-1.41) | 0.699 |
| ASA score >2 | 0.84 (0.58-1.22) | 0.359 | 0.72 (0.47-1,10) | 0.126 |
| Hyperbilirubinaemia | 0.59 (0.22-1.55) | 0.282 | 0.54 (0.19-1.58) | 0.263 |
| Cholangitis | 0.59 (0.23-1.56) | 0.289 | 0.59 (0.20-1.61) | 0.287 |
| Anemia | 0.74 (0.51-1.08) | 0.121 | 0.65 (0.42-0.99) | **0.046** |
| ASA: American society of anesthesiologists; BMI: body mass index; CI: confidence interval; COPD: chronic obstructive pulmonary disease; OR: odds ratio; UICC: Union for International Cancer Control | | | | |

| **Supplementary table 4B** Uni- and multivariable analysis of intraoperative factors for textbook outcome in CRLM | | | | |
| --- | --- | --- | --- | --- |
| **Variables** | univariable | | multivariable | |
|  | OR (CI) | p-value | OR (CI) | p-value |
| Left or extended left versus  right or extended right hepatectomy | 1.17 (0.76-1.82) | 0.477 | 1.12 (0.71-1.74) | 0.634 |
| Biliary procedure | 1.35 (0.59-3.11) | 0.479 | 1.64 (0.67-4.05) | 0.282 |
| Minimally invasive procedure | 0.89 (0.59-1.35) | 0.593 | 1.01 (0.65-1.57) | 0.957 |
| Vascular procedure^*^ | 1.08 (0.53-2.22) | 0.828 | 1.07 (0.50-2.26) | 0.871 |
| Pringle maneuver | 0.98 (0.68-1.41) | 0.910 | 0.94 (0.64-1.36) | 0.731 |
| Two-staged hepatectomy | 0.66 (0.25-1.75) | 0.399 | 0.70 (0.25-1.97) | 0.498 |
| Multivisceral resection^+^ | 0.59 (0.35-1.00) | 0.052 | 0.66 (0.38-1.14) | 0.135 |
| Emergency procedure | 0.47 (0.09-2.60) | 0.387 | 0.39 (0.07-2.31) | 0.298 |
| Operation time >286 minutes | 0.56 (0.38-0.81) | **0.002** | 0.57 (0.38-0.84) | **0.005** |
| CI: confidence interval; OR: odds ratio  ^*^Vascular procedure: reconstructions of portal vein, inferior cava vein or hepatic artery  ^+^Multivisceral resection: resection of stomach, pancreas, colon, rectum or diaphragm | | | | |
